# Intraseasonal waning of influenza vaccine effectiveness and implications for the optimal timing of seasonal vaccination programs: A scoping review

**DOI:** 10.14745/ccdr.v52i06a03

**Published:** 2026-06-30

**Authors:** Pamela Doyon-Plourde, Fazia Tadount, Anabel Gil, Calin Lazarescu, Marie-Michelle Ursu, Nadine Sicard, Winnie Siu, Angela Sinilaité

**Affiliations:** 1 Centre for Immunization Surveillance and Programs, Public Health Agency of Canada, Ottawa, ON; 2 Département de microbiologie, infectiologie et immunologie, Université de Montréal, Montréal, QC; 3 School of Epidemiology and Public Health, Faculty of Medicine, University of Ottawa, Ottawa, ON

**Keywords:** influenza vaccine, duration of protection, vaccine effectiveness, waning, optimal timing

## Abstract

**Background:**

Seasonal influenza vaccination programs aim to provide protection before the virus circulation begins. However, vaccine effectiveness (VE) may decline within a single influenza season due to waning immunity, antigenic drift, and season-specific factors, raising questions about optimal vaccination timing. The purpose of this review was to synthesize evidence on intraseasonal waning of seasonal influenza VE and examine the implications and key considerations for optimizing the timing of seasonal vaccination in Canada.

**Methods:**

A scoping review was conducted, with searches of MEDLINE, Embase, Scopus, Cochrane Library, and ProQuest Public Health Database that identified studies published from 2010 to June 2024, with an update in July 2025. Eligible studies included clinical trials, observational studies, systematic reviews, and modelling studies reporting at least two VE estimates by time since vaccination within a season or assessing vaccination timing and its impact on influenza outcomes.

**Results:**

Forty-nine studies met inclusion criteria, including 37 assessing intraseasonal waning and 12 examining vaccination timing. Overall, VE was generally highest within one to three months post-vaccination and declined over the season (e.g., three to six months post-vaccination). Waning was more consistently observed for influenza A, particularly A(H3N2), and among adults aged 60 years and older. Modelling studies suggested that delaying vaccination could reduce influenza burden under select late-peaking or fast-waning scenarios; however, estimated benefits were generally small and highly sensitive to assumptions.

**Conclusion:**

Intraseasonal waning of influenza VE is consistently observed, particularly for influenza A and in older adults, but represents a gradual decline that does not typically undermine season-long effectiveness. Evidence does not support substantial or consistent population-level benefits from delaying vaccination.

## Introduction

Seasonal influenza in Canada follows a predictable annual pattern, typically beginning in December and lasting 12–16 weeks, although seasons may start as early as October or as late as February and can extend up to 20 weeks (1). Influenza activity generally progresses from west to east across the country (1). Annual vaccination is the primary strategy for preventing influenza and its complications, and in Canada and other northern hemisphere countries, immunization programs typically begin in October to ensure protection before virus circulation increases (1–3).

While fall vaccination is well established as the recommended timing for seasonal influenza immunization, research continues to examine how vaccination timing may influence effectiveness across the influenza season. Emerging evidence indicates that vaccine effectiveness (VE) may decline within a season due to waning immunity, prior immunity, underlying health conditions, and changes in circulating strains (4–6). Observational studies have reported increasing odds of laboratory-confirmed influenza (LCI) with longer time since vaccination, particularly for A(H3N2), raising concerns that early vaccination could result in reduced protection later in the season, especially among populations at higher risk of severe outcomes (7).

Beyond waning immunity, VE and vaccination impact are influenced by season-to-season variability in influenza circulation, antigenic drift, vaccine-strain match, and programmatic factors, such as supply and uptake (8).

While prior reviews have examined intraseasonal waning, an integrated assessment that jointly considers waning and evidence informing vaccination timing has been lacking. This scoping review addresses this gap by mapping the available evidence and synthesizing insights to support evidence-based public health decision-making and ongoing national work to optimize influenza vaccination strategies in Canada. The objectives were to: i) summarize recent evidence on intraseasonal waning of protection conferred by seasonal influenza vaccines; and ii) identify key considerations related to the optimal timing of seasonal influenza vaccination.

## Methods

This scoping review followed the Joanna Briggs Institute methodological framework and is reported in accordance with the Preferred Reporting Items for Systematic Reviews and Meta-Analyses extension for scoping reviews (PRISMA-ScR) (9,10). The protocol was registered on the Open Science Framework (OSF ID: Rkzc9) (11).

### Search strategy

A comprehensive search strategy was developed with a research librarian and applied to MEDLINE, Embase, Scopus, Cochrane Library, and the ProQuest Public Health Database (**Appendix**, Supplemental material, Tables S1–S8). Searches included English and French language publications from January 1, 2010 (post-2009 H1N1 strain introduction) to June 17, 2024, and were updated on July 15, 2025.

### Eligibility criteria

Studies included individuals six months of age and older in temperate regions of the northern or southern hemisphere, as well as Australia and New Zealand, due to their research quality and mostly having a temperate climate. Eligible study designs included primary studies (i.e., clinical trials and observational studies), systematic reviews, and meta-analyses.

For evidence on waning protection conferred by seasonal influenza vaccination, studies were required to report two VE estimates by time since vaccination within a single influenza season, using LCI or influenza-related hospitalization outcomes. For evidence related to optimal timing of influenza vaccination, eligible studies assessed vaccination timing through comparisons of earlier versus later vaccine administration in relation to influenza outcomes or modelled the impact of vaccination timing on influenza-related outcomes.

Studies of pandemic monovalent or investigational vaccines, immunogenicity-only outcomes, relative VE without absolute estimates, non-human studies, and studies focused solely on vaccination coverage or uptake were excluded.

### Study selection and data extraction

Citations were imported into Zotero for deduplication and uploaded to DistillerSR (Evidence Partners Inc, Ottawa, Canada) for screening. Titles and abstracts were screened by three independent reviewers. A record was included if at least one reviewer deemed it potentially eligible; exclusion required agreement by two reviewers. The DistillerSR artificial intelligence (AI) review tool was used to support exclusion at title and abstract level using a conservative relevance threshold of 0.2 (scores near 0 indicating high-confidence exclusion). All AI-flagged exclusions were manually reviewed. A record was excluded only when at least one reviewer agreed with the AI’s recommendation. Disagreements were resolved through discussion. Records not flagged by AI underwent standard dual human screening. Full-text screening was conducted by two independent reviewers, with discrepancies resolved through discussion.

Data extraction was completed by one reviewer and validated by a second, capturing study characteristics (e.g., population, setting, design), interventions, outcomes, and findings relevant to each objective.

### Risk of bias assessment

Risk of bias was assessed to provide context for policy interpretation. Critical appraisal was conducted by one reviewer and validated by a second reviewer. Tools included the revised Cochrane Risk of Bias Tool for randomized trials (12), the Risk of Bias in Non-randomized Studies of Interventions (13,14) and A MeaSurement Tool to Assess Systematic Reviews (AMSTAR 2) (15). Modelling studies were not assessed for risk of bias.

### Evidence synthesis

Findings were summarized narratively and VE estimates stratified by time since vaccination were presented graphically in a forest plot. Findings related to optimal timing of influenza vaccination were synthesized separately, highlighting key assumptions and factors influencing timing decisions.

## Results

**Figure 1:**
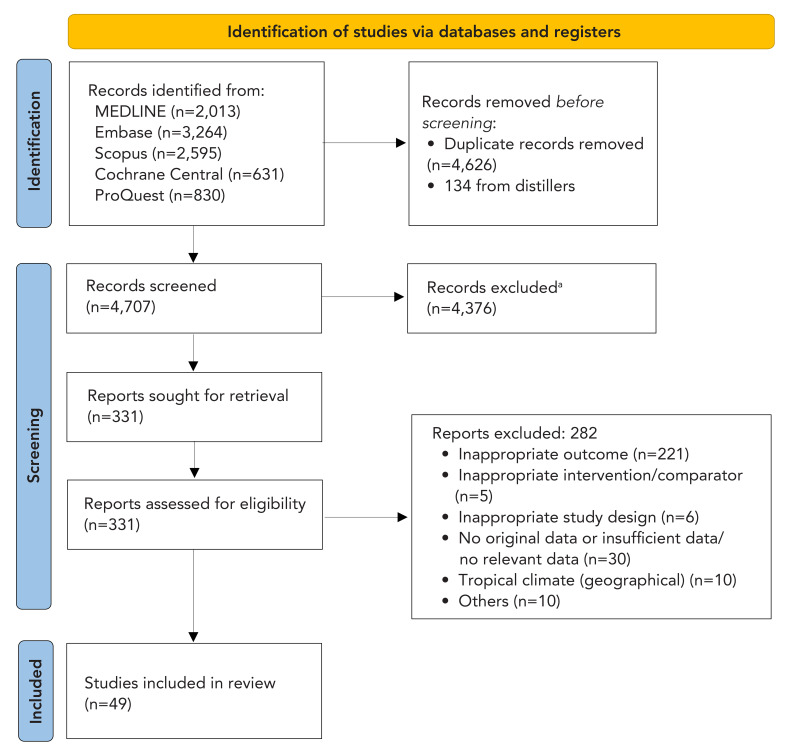
PRISMA diagram^a^ ^a^ Level 1 (title/abstract) screening in DistillerSR used a dual configuration (human reviewer + AI reviewer). The AI was restricted to suggesting potential exclusions only. All AI-suggested exclusions (n=3,242) were reviewed and confirmed by a human reviewer

Overall, 49 studies met inclusion criteria (**Figure 1**), covering influenza seasons from 2005–2006 to 2023–2024 across multiple countries (**Table 1** and **Table 2**). Most studies used a test-negative design (TND) and assessed intraseasonal waning of VE against LCI.

**Table 1:** Characteristics of primary research studies (observational studies and clinical trials)

Author, year	Country, season	Study design	Study population (n) setting	Intervention (n)	Control (n)	Outcome	RoB
**Intraseasonal waning of seasonal influenza vaccine protection**							
Castilla *et al.*, 2013	Spain 2011–2012	TND	Individuals (≥6 months), excluding HCWs, and nursing home residents (n=757) Hospitals-regional influenza surveillance	IIV3-SD (n=193)	Unvaccinated (n=564)	LCI hospitalization (PCR)	Low^a^
Jimenez-Jorge *et al.*, 2013	Spain 2011–2012	TND	Older adults (≥65 years old) and individuals <65 years old at high risks with ILI (n=378) Primary care	IV (n=134)	Unvaccinated (n=208)	LCI (PCR and/or culture)	Low^a^
Kissling *et al.*, 2013	France, Hungary, Ireland, Italy, Poland, Portugal, Romania and Spain 2011–2012	TND	Non-institutionalized adults (≥18 years old) with ILI or ARI (n=1,016) Primary care and hospitals part of the sentinel networks	IV (n=367)	Unvaccinated (n=649)	LCI (PCR or culture)	Low^a^
Pebody *et al.*, 2013	United Kingdom 2011–2012	TND	Individuals with ILI (n=3,869) Primary care	IIV3 (n=745)	Unvaccinated (n=2,954)	LCI (PCR)	Low^a^
Andrews *et al.*, 2014	United Kingdom 2012–2013	TND	Individuals (≥6 months) with ILI (n=3,286) Primary care	LAIV3 (n=534)	Unvaccinated (n=2,752)	LCI (PCR)	Low^a^
Sullivan *et al.*, 2014	Australia 2012	TND	Individuals with ILI (n=600) Primary care	IIV3 (n=134)	Unvaccinated (n=466)	LCI (PCR)	Low^a^
Pebody *et al.*, 2015	United Kingdom 2014–2015	TND	Individuals (≥6 months) with ILI (n=2,931) Primary care	LAIV (children) or IIV (n=732)	Unvaccinated (n=2,199)	LCI (PCR)	Low^a^
Gherasim *et al.*, 2016	Spain 2014–2015	TND	Individuals with ILI (n=5,044) Primary care and hospitals part of the sentinel networks	IIV3 (n=520)	Unvaccinated (n=4,524)	LCI (PCR)	Low^a^
Kissling *et al.*, 2016	Germany, Spain, France, Hungary, Ireland, Italy, Poland, Portugal, and Romania 2010–2011 to 2014–2015	TND	Individuals with ILI (n=23,167) Primary care	IIV (n=2,224)	Unvaccinated (n=20,943)	LCI (PCR)	Low^a^
Pebody *et al.*, 2016	United Kingdom 2015–2016	TND	Individuals with acute ILI (n=3,841) Primary care	LAIV (children) or IIV4 (n=892)	Unvaccinated (n=2,949)	LCI (PCR)	Low^a^
Radin *et al.*, 2016	United States 2010–2011 to 2013–2014	TND	Individuals with febrile RI (n=1,481) Outpatient health care	IIV and LAIV (n=612)	Unvaccinated (n=869)	LCI (PCR)	Moderate^a^
Ferdinands *et al.*, 2017	United States 2011–2012 to 2014–2015	TND	Individuals (≥9 years old) with ARI (n=20,825) Outpatient setting	IV (n=9,094)	Unvaccinated (n=11,731)	LCI (PCR)	Low^a^
Hergens *et al.*, 2017	Sweden and Finland 2016–2017	TND	Older adults (≥65 years old) living in Sweden (n=358,583) or Finland (n=1,144,894) Primary care and hospitals	IIV (Stockholm n=157,477, Finland n=532,076)	Unvaccinated (Sweden n=201,106, Finland n=612,818)	LCI	Low^a^
Bi *et al.*, 2024	United States 2011–2012 to 2018–2019	TND	Individuals (≥6 months) with ARI (n=55,728) Outpatient care	IIV or LAIV (n=27,986)	Unvaccinated (n=27,742)	LCI (PCR)	Moderate^a^
Chiu *et al.*, 2018	China 2016–2017	TND	Children (6 months–17 years old) with ARI (n=5,514) Hospitals	IIV3 or IIV4 (n=495)	Unvaccinated (n=5,019)	LCI hospitalization (PCR)	Moderate^a^
Feng *et al.*, 2018	China 2012–2013 to 2016–2017	TND	Children (6 months–17 years) with hospitalized for RI (n=15,695) Hospitals	LAIV3 or LAIV4 (n=1,604)	Unvaccinated (n=14,091)	LCI (DIF assay, culture, PCR)	Moderate^a^
Young *et al.*, 2018	Europe, Kenya, Thailand, Australia 2009–2016	SR-MA of TND (n=14)	Individuals with ARI Primary care facilities (n=11)	IV (NR)	Unvaccinated (NR)	LCI (PCR)	Moderate^c^
Pebody *et al.*, 2019	United Kingdom 2017–2018	TND	Individuals with acute ILI (n=3,080) Primary care	LAIV4 (children), IIV3 or IIV4 (n=838)	Unvaccinated (n=2,242)	LCI (PCR)	Low^a^
Powell *et al.*, 2019	United States 2017–2018	TND	Children (6 months–17 years old) with ARI (n=3,595) Hospitals	IV (n=632)	Unvaccinated (n=3,063)	LCI hospitalization (RIT or PCR)	Low^a^
Ray *et al.*, 2019	United States 2010–2011 to 2016–2017	TND	Vaccinated individuals (≥2 years old) tested for influenza virus (n=49,272 person-year) Primary care and hospitals	IIV (n=45,184)	Time since vaccination compared to reference period (14–41 days)	LCI (PCR)	Low^a^
Regan *et al.*, 2019	Australia 2016	TND	Individuals with ILI (n=2,085) Primary care	IV (n=332)	Unvaccinated (n=1,753)	LCI (PCR)	Serious^a^
Wang *et al.*, 2020	China 2016–2017	RCT phase 3	Healthy children 3–17 years old (n=1,999) Primary care and hospitals	LAIV3 (n=996)	Placebo (n=996)	LCI (PCR)	Low^b^
Ferdinands *et al.*, 2021	United States 2015–2016 to 2018–2019	TND	Adults (≥18 years old) hospitalized for ARI (n=3,016 A(H3N2), 1,492 A(H1N1)pdm09, and 1,060 B/Yamagata) Hospital-based	IV (n=3,589)	Unvaccinated (n=1,979)	LCI hospitalization (PCR)	Low^a^
Mira-Iglesias *et al.*, 2021	Spain 2018–2019	TND	Older adults (≥65 years old) with ILI (n=992) Primary care and hospitals	IIV3 or IIV3-Adj (n=662)	Unvaccinated (n=330)	LCI hospitalization (PCR)	Low^a^
Sahni *et al.*, 2021	United States 2015–2016 to 2019–2020	TND	Children (≥6 months–<18 years old) with ARI (n=8,430) Hospitals	IV (n=4,653)	Unvaccinated (n=3,777)	LCI hospitalization (PCR)	Low^a^
Hu *et al.*, 2022	United States 2016–2017 to 2019–2020	TND	Adults (≥18 years old) with ILI (n=7,114) Outpatient care	IIV-SD (n=4,071)	Unvaccinated (n=3,043)	LCI (PCR)	Low^a^
Tenforde *et al.*, 2023	United States 2021–2022	TND	Adults (≥18 years) with visits or hospitalization for ARI (n=86,732) Hospitals (emergency department)	IV (n=45,136); approximately 5,879 had IIV-HD, 10,559 had IIV-SD and 11,721 had IIV-Adj	Unvaccinated (n=58,401)	LCI (NR)	Low^a^
Chung *et al.*, 2024	Canada 2010–2011 to 2018–2019	TND	Vaccinated individuals (≥6 months, n=53,065) Hospital-based	LAIV3 or LAIV4 (n=53,065)	Time since vaccination compared to reference period (14–41 days)	LCI (PCR)	Low^a^
Domnich *et al.*, 2024	Italy 2018–2019 to 2022–2023	TND	Individuals (≥6 months) with ILI or hospitalized for SARI (n=6,490) Primary care or hospitals	IIV-SD (Mid-October to November, n=1,571)	Unvaccinated (n=4,857)	LCI (PCR)	Low^a^
Lewis *et al.*, 2024	United States 2022–2023	TND	Adults (≥18 years old) with (n=3,707) ARI Hospitals	IIV (n=1,697), older adults likely received IIV-HD or IIV-Adj	Unvaccinated (n=2,010)	LCI hospitalization (PCR)	Low^a^
Maurel *et al.*, 2024	Croatia, France, Germany, Hungary, Ireland, the Netherlands, Portugal, Romania, Spain national, Spain Navarra region and Sweden 2022–2023	TND	Individuals with ARI or ILI (n=38,058) Primary care	IV (n=6,380)	Unvaccinated (n=31,678)	LCI (PCR)	Low^a^
Seppälä *et al.*, 2024	Norway 2022–2023	Retrospective cohort study	Older adults (≥65 years) with SARI (n=1,005,568) Hospitals	IIV4 or IIV4-Adj (n=637,827)	Unvaccinated (n=367,741)	Influenza-associated hospitalisation and death (ICD-10 codes J09, J10 or J11)	Low^a^
Zhang *et al.*, 2024	China 2022–2023	TND	Individuals (≥6 months) with ILI (n=8,301) Hospital-based	IIV3-SD or IIV4-SD (n=182)	Unvaccinated (n=8,119)	LCI hospitalization (PCR)	Low^a^
Zhu *et al.*, 2024	China 2021–2022 to 2023–2024	TND	Children (6 months–<18 years old) with ARI (n=27,670) Outpatient setting	IIV-SD or LAIV (n=2,857)	Unvaccinated (n=24,813)	LCI (PCR or culture)	Moderate^a^
Abou Chakra *et al.*, 2025	France 2023–2024	TND	Individuals with ILI (n=146,662) Primary care	IIV4-SD or IIV4-HD (n=32,740)	Unvaccinated (n=113,922)	LCI (PCR)	Moderate^a^
Lewis *et al.*, 2025	United States 2023–2024	TND	Adults (≥18 years old) hospitalized with ARI (n=7,690) Hospital-based	IV (n=3,170)	Unvaccinated (n=4,520)	LCI hospitalization (PCR)	Low^a^
Zhu *et al.*, 2025	United States 2023–2024	TND	Individuals (≥6 months) with ARI (n=1,382,142) Outpatient and hospitals	IV (n=415,390); of adults ≥65 years, 84,739 had IIV-HD, 1,260 had RIV and 64,959 IIV-Adj	Unvaccinated (n=966,752)	LCI (PCR)	Moderate^a^
**Optimal timing of influenza vaccine administration**							
Glinka *et al.*, 2016	United States 2005–2006 to 2012–2013	Retrospective cohort study	HIV-positive adults in care; vaccinated predominantly with IIV-SD (n=1,176 HIV-positive adults; 4,575 vaccination events across seasons) Outpatient setting	IIV-SD Early scenario: September 1–November 15	Late scenario: after November 15	Incidence of LCI or ILI	Serious^a^
Worsham *et al.*, 2024	United States 2011–2012 to 2017–2018	Retrospective cohort study	Children vaccinated between August 1 and January 31 across seasons (n=819,223 children; 1,261,164 child-seasons) MarketScan commercial insurance claims database	Vaccination timing driven by birth month (children born in October more likely vaccinated in October)	Cross comparison across birth-month groups representing earlier versus later vaccination timing (August vs October; August vs December; October vs December)	Rate of influenza diagnosis by ICD codes or oseltamivir claim (ICD-9 or ICD-10)	Low^a^

Abbreviations: ARI, acute respiratory illness; DIF, direct immunofluorescence; HCWs, healthcare workers; ICD, International Classification of Diseases; ICD-9, International Classification of Diseases, Ninth Revision; ICD-10, International Classification of Diseases, Tenth Revision; IIV, inactivated influenza vaccine; IIV-Adj, adjuvanted inactivated influenza vaccine; IIV-HD, high-dose inactivated influenza vaccine; IIV-SD, standard-dose inactivated influenza vaccine; IIV3, trivalent inactivated influenza vaccine; IIV3-Adj, adjuvanted trivalent inactivated influenza vaccine; IIV3-SD, standard-dose trivalent inactivated influenza vaccine; IIV4, quadrivalent inactivated influenza vaccine; IIV4-Adj, adjuvanted quadrivalent inactivated influenza vaccine; IIV4-HD, high-dose quadrivalent inactivated influenza vaccine; IIV4-SD, standard-dose quadrivalent inactivated influenza vaccine; ILI, influenza like-illness; IV, influenza vaccine; LAIV, live attenuated influenza vaccine; LAIV3, trivalent live attenuated influenza vaccine; LAIV4, quadrivalent live attenuated influenza vaccine; LCI, laboratory-confirmed influenza; NR, not reported; PCR, polymerase chain reaction; RCT, randomized controlled trial; RI, respiratory illness; RIT, rapid influenza test; RIV, recombinant influenza vaccine; RoB, risk of bias; SARI, severe acute respiratory illness; SR-MA, systematic review and meta-analysis; TND, test-negative design

^a^ Risk of bias was evaluated using the Risk of Bias in Non-randomized Studies of Intervention tool (ROBINS-I)

^b^ Risk of bias was evaluated using the revised Cochrane Risk-of-Bias tool for randomized trials (ROB 2.0)

^c^ Risk of bias was evaluated using the A Measurement Tool to Assess Systematic Reviews tool for systematic review (AMSTAR 2)

**Table 2:** Key characteristics and findings of modelling studies evaluating optimal timing for influenza vaccination

Author, year	Population setting	Timing scenarios compared	Key assumptions driving results	Key result relevant to timing
Lee *et al.*, 2010	Children, United States	September–October vs later	Early-season protection emphasized	September–October vaccination most cost-effective
Myers *et al.*, 2010	Pregnant individuals, United States	Early vs delayed (>November)	No explicit waning	Delaying vaccination reduced effectiveness and cost-effectiveness
Lee *et al.*, 2015	General population, United States	Earlier vs current practice	Season timing and variability	Earlier vaccination yielded modest savings; increasing coverage had larger impact
Newall *et al.*, 2018	Adults 65 years of age and older, United States	August–November	Fast vs slow waning; season variability	Optimal timing varied by season and waning rate
Costantino *et al.*, 2019	General population, Australia	Early vs delayed (by ~2–3 months)	Waning; coverage held constant vs reduced	Delayed vaccination modestly improved outcomes only if coverage remained stable; benefits lost with small coverage declines
Smith *et al.*, 2019	Adults 65 years of age and older, United States	Compressed (October–May) vs extended (August–May) season	Waning; coverage loss; season variability	Small benefit of compression offset by reduced uptake
Ferdinands *et al.*, 2020	Adults 65 years of age and older, United States	August–September vs October	Waning; influenza season timing; VE; coverage loss with delay	Delaying vaccination increased hospitalization if more than 14% missed vaccination
Kahana *et al.*, 2021	General population, Israel	Program start dates from July 1 to December 1 (e.g., September vs October start)	Coverage-dependent rollout speed; waning	Optimal timing shifted later as coverage increased
Williams *et al.*, 2022	Adults 65 years of age and older	One-dose vs two-dose; October start	Waning, second-dose uptake	Benefits depended on late peaks and high uptake
Spencer *et al.*, 2024	Multiple age groups	Early vs delayed	Initial VE, waning rate, peak timing, coverage held constant	Optimal timing highly sensitive to waning and peak timing

Abbreviation: VE, vaccine effectiveness

### Intraseasonal waning of seasonal influenza vaccine protection

Thirty-seven studies assessed intraseasonal waning, including 34 TND studies, one cohort study, one randomized controlled trial (RCT), and one systematic review and meta-analysis (Table 1).

The RCT was assessed as low risk of bias (Table 1). Most non-randomized studies were rated low (n=27) to moderate (n=7) risk of bias, with one at serious risk. The systematic review received a moderate confidence rating. Common limitations included residual confounding (e.g., health status) and missing data.

### Overall population-level estimates

**Figure 2:**
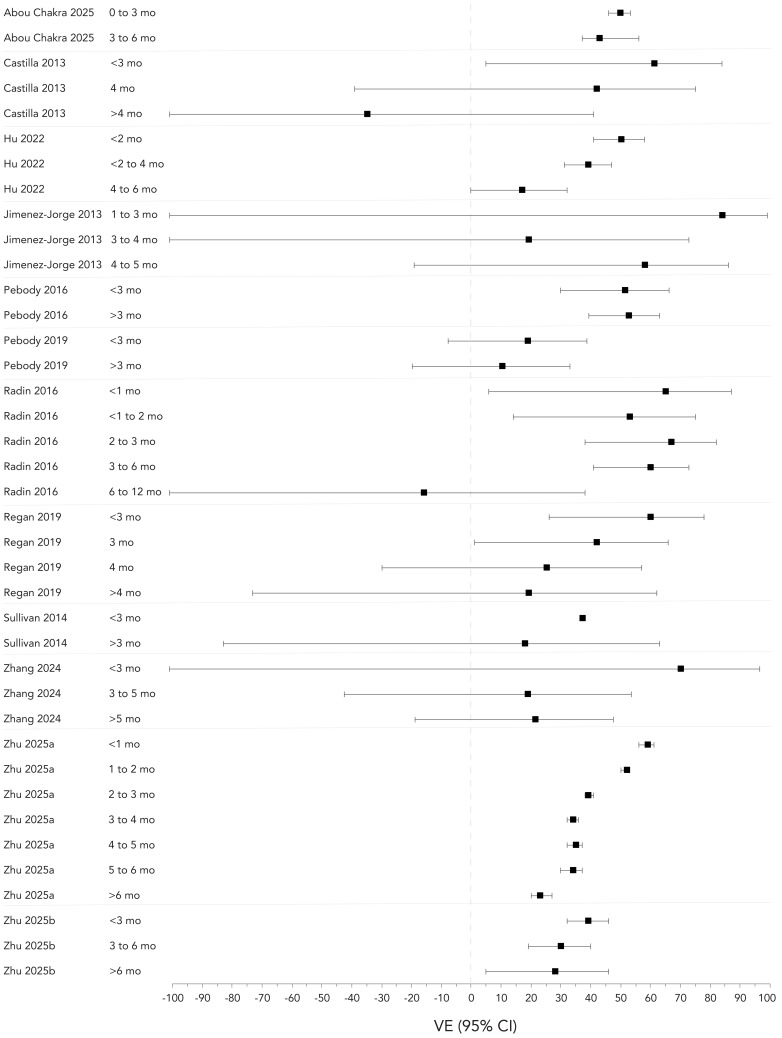
Influenza vaccine effectiveness against laboratory-confirmed infection by time since vaccination Abbreviations: CI, confidence interval; mo, months; VE, vaccine effectiveness

Across 28 studies reporting population-level estimates without age stratification, VE was highest shortly after vaccination and generally declined over the course of the influenza season (**Figure 2**) (4,7,16–41). Waning was more consistently observed for influenza A than for influenza B, particularly A(H3N2) (Supplemental material, Table S9). For LCI, most studies reported waning within three to six months post-vaccination, although magnitude varied; one study reported no evidence of decline (Figure 2) (30).

Three studies evaluated VE against influenza-related hospitalization (25,26,36). Two reported declining VE with increasing time since vaccination, with reductions of approximately 20%–35% beyond 120 days (26,36), while one observed relatively stable VE (~39%) with wider confidence intervals (CIs) later in the season (25). Strain-specific estimates from Lewis *et al.* showed greater stability for influenza B than influenza A, with VE declining from 72% to 62% for influenza B and from 42% to 16% for influenza A across comparable time intervals (26).

A meta-analysis by Young *et al.*, which included studies also captured in this review, reported significant pooled declines in VE between early (15–90 days) and later (91–180 days) intervals for influenza A(H3N2) (∆VE=−33%, 95% CI: −57%–−12%, n=11 studies) and influenza B (∆VE=−19%, 95% CI: −33%–−6%, n=6 studies), but not for A(H1N1) (∆VE=−8%, 95% CI: −27%–21%, n=5 studies) (7). These results aligned with patterns observed in individual studies.

Analyses modelling time since vaccination as a continuous variable further supported waning. Ray *et al.* reported a 16% increase in the odds of influenza infection per 28 days post-vaccination (odds ratio [OR]: 1.16, 95% CI: 1.13%–1.20%) (33). Ferdinands *et al.* estimated average VE declines per 30 days of 7.5% (95% CI: 0.3%–16.3%) for A(H3N2), 8.5% (95% CI: 3.0%–17.0%) for A(H1N1), and 8.0% (95% CI: 1.4%–21.9%) for influenza B/Yamagata among hospitalized adults, with similar findings for infection outcomes reported by Ferdinands *et al.* (40,41).

Overall, population-level evidence indicated progressive intraseasonal waning, with more consistent evidence available for LCI than for hospitalization outcomes, and more pronounced for influenza A than for influenza B.

### Children

Eight studies reported paediatric-specific VE estimates (4,16,42–47). Overall, VE was highest shortly after vaccination and generally remained stable or declined modestly over the season. For LCI, early–late differences were small, with changes in VE of approximately 5%–8% across the season (4,16,42,43) (Supplemental material, Table S10).

Two studies assessed influenza-related hospitalization in children and reported VE declines of approximately 20% and 30% within six or nine months post-vaccination (44,45). Age-stratified analyses showed broadly similar patterns across paediatric age groups (e.g., <2 years, 3–5 years, and 6–17 years), with modest seasonal variation for LCI and larger declines observed for hospitalization outcomes (16,44,45).

Two studies reported increasing odds of influenza positivity with time since vaccination (46,47), although significant waning was observed for A(H3N2) in only one study (46). Overall, VE in children remained protective throughout the season, with limited evidence of substantial intraseasonal waning.

### Adults 18 to 64 years of age

Three studies specifically assessed intraseasonal waning of influenza in adults aged 18–64 years (4,16,46). In general, VE was highest shortly after vaccination, with variable changes over time. One study reported a modest decline (~5%) at three to six months, another reported larger declines (~30%) beyond five months, and a third found no significant association between time since vaccination and influenza positivity (adjusted OR: 1.00; 95% CI: 0.95%–1.06%) (4,16,46).

Overall, evidence in this age group was limited and heterogeneous, precluding firm conclusions regarding the extend of intraseasonal waning.

### Older adults

Ten studies evaluated intraseasonal waning in adults 60 or 65 years and older (4,16,19,22,24,40,46,48–50). Most reported declining VE over time, with patterns varying by outcome and strain. For LCI, VE was generally highest within one to three months post-vaccination and declined thereafter (**Table 3**), with steeper waning for A(H3N2) than influenza B in one study (24).

**Table 3:** Influenza vaccine effectiveness against laboratory-confirmed influenza by time since vaccination in older adults 60 years of age and older

Study	Age groups	Time since vaccination	VE (95% CI)
Abou Chakra *et al.*, 2025	65 years and older	15 days–3 months	43.11% (37.07–48.61)
		3–6 months	39.71% (31.31–47.19)
Castilla *et al.*, 2013	65 years and older	<100 days	47% (−63–83)
		100–119 days	54% (−55–86)
		≥120 days	32% (−111–78)
Chung *et al.*, 2024	65 years and older	42–69 days	−7% (−26–9)
		70–97 days	−17% (−41–3)
		98–125 days	−30% (−60–−5)
		126–153 days	−32% (−67–−4)
		≥154 days	−32% (−78–3)
Jimenez-Jorge *et al.*, 2013	65 years and older	49–88 days	85% (18–97)
		89–127 days	33% (−102–78)
		128–166 days	−376% (−4,332–49)
Kissling *et al.*, 2016^a^	60 years and older	40 days	A(H3N2): 45% (7–67)
		80 days	A(H3N2): 33% (12–49)
		120 days	A(H3N2): 10% (−18–32)
		160 days	A(H3N2): −16% (−93–31)
		40 days	Influenza B: 62% (3–84)
		80 days	Influenza B: 55% (32–71)
		120 days	Influenza B: 41% (13–60)
		160 days	Influenza B: 24% (−39–58)

Abbreviations: CI, confidence interval; VE, vaccine effectiveness

^a^ Vaccine effectiveness estimated from plots

Hospitalization outcomes showed similar temporal patterns, often with less pronounced declines and widening CIs later in the season. Mira-Iglesias *et al.* reported VE decreasing from 54% at 15–82 days to 50% at 123–177 days post-vaccination (48). In Seppälä *et al.*, VE among adults 65–79 years declined from 34% at 7–89 days to −3% beyond 180 days, whereas VE remained around 40% in adults aged 80 years and older, with CI widening over time (49).

Continuous-time analyses generally supported waning (40,46,50). Domnich *et al.* reported a significant 6%–7% increase in odds of influenza per week since vaccination for any influenza and A(H3N2), starting 14 days post-vaccination (46). Ferdinands *et al.* estimated average declines in VE of approximately 10% per 30 days post-vaccination for hospitalization (40). However, another study observed seasonal declines in VE, but sensitivity analyses accounting for calendar week and time since vaccination did not demonstrate a clear gradual decline, with overlapping CIs across weekly VE estimates (50).

Overall, VE in older adults was highest shortly after vaccination and declined over the course of the season, with considerable heterogeneity across studies.

### Optimal timing of influenza vaccine administration

Twelve studies assessed optimal vaccination timing, including ten modelling studies and two observational studies (Table 1 and Table 2) (51–62). Modelling studies evaluated vaccination timing under varying assumptions related to VE, intraseasonal waning, season timing, and vaccination coverage (Table 2), while observational studies compared influenza outcomes following earlier versus later vaccination in specific population (Table 1).

Modelling results suggested that under some late-peaking or fast-waning scenarios, delaying vaccination by one to two months could reduce influenza burden when coverage was held constant (51–53,57,58,60). However, the estimated gains were generally small, typically corresponding to less than a 5% difference in prevented cases or hospitalizations between timing scenarios (51,57,58). Several models further showed that even modest reductions in vaccination coverage associated with delayed uptake could offset or reverse these potential benefits (51,52,58). Economic modelling favoured earlier fall vaccination, particularly for children and pregnant individuals (54–56). Models of compressed vaccination periods (e.g., October–May vs August–May) or two-dose schedules in older adults showed benefits under select late-peaking or fast-waning scenarios, which diminished when coverage or adherence declined (58,59).

Two observational studies reported mixed findings. Glinka *et al.* observed higher influenza-related outcomes among HIV-infected individuals vaccinated earlier (before mid-November) compared with later (after mid-November) vaccination (61). In contrast, a population-based cohort study in young children found that those born in October, who were disproportionately vaccinated in October, were least likely to receive an influenza diagnosis, particularly compared with children born in August, who tended to be vaccinated earlier, and those born in December, who tended to be vaccinated later (62). Both studies were subject to potential residual confounding.

Overall, modelling evidence suggests that the impact of modifying vaccination timing is highly sensitive to assumptions about waning, season timing, and vaccination coverage, with potential gains from delayed strategies generally small and easily negated by reductions in uptake, while observational evidence remains limited and inconsistent.

## Discussion

This scoping review synthesized evidence on intraseasonal waning of influenza VE and its implications for seasonal vaccination timing, addressing gaps identified in recent national evidence syntheses. Across age groups and study designs, VE was generally highest within one to three months after vaccination and declined progressively over the influenza season (e.g., 3–6 months post-vaccination). Waning was most consistently observed for influenza A, particularly A(H3N2), and among older adults. Evidence for hospitalization outcomes was more limited than for LCI, but available studies suggested similar temporal patterns with greater uncertainty.

These findings align with earlier evidence syntheses, including the 2017 meta-analysis by Young *et al.* and a prior systematic review and meta-analysis of immunogenicity and antibody persistence, which demonstrated biologically plausible declines in vaccine-induced immunity by six months post-vaccination (6,63). However, the magnitude and consistency of waning varied across studies, likely reflecting differences in circulating strains, season timing, population characteristics, and analytic approaches. Importantly, observed late season declines may reflect not only waning immunity but also antigenic drift and reduced vaccine-strain match, particularly during A(H3N2)-dominant seasons (64).

Evidence on vaccination timing suggested that potential benefits of delaying vaccination are context-dependent and generally modest. Modelling studies indicated that in specific scenarios (e.g., late-peaking seasons or rapid waning), later vaccination could reduce influenza burden when coverage was held constant (51–53,57,58,60). However, projected gains were small and highly sensitive to assumptions about waning, season timing, and uptake, with several models showing that modest coverage reductions could negate benefits (51,52,58). Observational studies examining vaccination timing reported mixed findings and were subject to residual confounding (61,62).

Overall, maintaining high vaccination coverage appears to be a dominant determinant of population-level impact. While waning is a consistent feature of influenza VE, the implications for modifying vaccination timing remain uncertain and vary by population, outcome, and season. Early-season vaccination provides protection across most seasons, whereas delayed strategies may leave individuals unprotected during early circulation, given variability in season onset and peak timing.

### Limitations

Interpretation should consider methodological limitations. Most studies relied on observational designs and were susceptible to residual confounding, time-varying biases and misclassification. Heterogeneity in definition of time since vaccination and adjustment for calendar time and circulating strains further complicates comparisons. Fewer studies assessed hospitalization outcomes, often with widening CIs later in the season. Modelling studies additionally depend on assumptions that may not reflect real-world conditions.

This review provides a comprehensive synthesis across multiple seasons, regions, age groups, and outcomes, integrating evidence on waning with vaccination timing. However, heterogeneity across studies, and limited data for certain populations, including pregnant individuals and immunocompromised persons, remain important gaps.

## Conclusion

Intraseasonal waning of influenza VE is consistently observed, particularly for influenza A and among older adults, but represents a gradual decline that does not typically undermine season-long effectiveness. Although timing adjustments may theoretically influence protection under specific scenarios, current evidence does not demonstrate substantial or consistent population-level benefits from modifying vaccination timing. These findings highlight the importance of considering waning alongside seasonal variability, strain dynamics, and programmatic factors, and underscore the need for further research on how these elements interact to influence influenza outcomes.

## Appendix

Supplemental material is available upon request to the author: naci-ccni@phac-aspc.gc.ca
